# Determinants of generic and specific health-related quality of life in patients with Parkinson’s disease

**DOI:** 10.1371/journal.pone.0178896

**Published:** 2017-06-26

**Authors:** Xiao-Jing Tu, Wen-Juh Hwang, Hui-Ing Ma, Ling-Hui Chang, Shih-Pin Hsu

**Affiliations:** 1 Institute of Allied Health Sciences, College of Medicine, National Cheng Kung University, Tainan, Taiwan; 2 Department of Neurology, College of Medicine, National Cheng Kung University, Tainan, Taiwan; 3 Department of Occupational Therapy, College of Medicine, National Cheng Kung University, Tainan, Taiwan; 4 Department of Neurology, E-Da Hospital and I-Shou University, Kaohsiung, Taiwan; Taipei Veterans General Hospital, TAIWAN

## Abstract

**Objectives:**

Generic and disease-specific health-related quality of life (HRQoL) instruments may reflect different aspects of lives in patients with Parkinson’s disease (PD) and thus be associated with different determinants. We used the same cluster of predictors for the generic and disease-specific HRQoL instruments to examine and compare the determinants of HRQoL.

**Method:**

HRQoL was measured in 92 patients with PD by the 36-item Short-Form Health Survey (SF-36) and the 39-item Parkinson’s Disease Questionnaire (PDQ-39). The predictors included demographic and disease characteristics, and motor and non-motor symptoms. Multiple regression analyses were used to identify HRQoL determinants.

**Results:**

Depressive symptoms and motor difficulties of daily living were the first two significant determinants for both instruments. The other significant determinant for the SF-36 was fatigue and non-motor difficulties of daily living, and for the PDQ-39 was motor signs of PD.

**Conclusions:**

The results suggest the importance of the evaluation and intervention focused on depressive symptoms and motor difficulties of daily living in patients with PD. In addition, the SF-36 seems more related to non-motor symptoms, while the PDQ-39 appears more associated with motor symptoms. This information is important for understanding results from these two instruments and for choosing which to use.

## Introduction

Parkinson’s disease (PD) is one of the most common age-related neurodegenerative disorders [1, 2]. PD is typically known for its cardinal symptoms: tremor, rigidity, movement slowness, and postural instability [1, 3, 4]. Recently there has been an increase in PD research and the non-motor symptoms such as depression and fatigue are also garnering attention [1, 2, 5]. The effects of disease can be evaluated at different domains and levels, from symptom-specific impairments of everyday functions to overall health-related quality of life (HRQoL) [1, 3]. The instruments used to measure the effects of a disease are important for guiding the development of treatment plans as well as for measuring improvements caused by interventions. With the rising awareness of client-centered practice, HRQoL measures have become essential for evaluating patients’ subjective well-being and overall health [6, 7]. Examining the determinants of HRQoL, usually at the symptom and function levels, provides critical information for setting priorities during the treatment planning stage and for reflecting changes important to patients in the outcome evaluation phase [1, 7]. Given that the effects of PD are multi-dimensional and widely varied, different HRQoL measures for the same group of patients might be associated with different determinants.

HRQoL measures can be categorized into two types: generic and disease-specific [3, 8–10]. Generic instruments are designed to summarize a wide scope of health concepts and can be used in populations with different diagnoses [3, 8–10]. In contrast, disease-specific instruments are designed for patients with a certain disease and focus on symptomatic problems of the disease [3, 8–10]. For PD, disease-specific HRQoL instruments are available (e.g., the 39-item Parkinson’s Disease Questionnaire [PDQ-39], Parkinson’s Impact Scale, and Parkinson’s Disease Quality of Life Questionnaire), and generic instruments are as well (e.g., the 36-item Short Form Health Survey [SF-36], Sickness Impact Profile, Nottingham Health Profile, and EuroQoL [EQ-5D]) [1, 10–12]. According to a systematic review on determinants of HRQoL in PD [5], the SF-36 is the most frequently used generic instrument, and the PDQ-39 is the most frequently used disease-specific instrument. The SF-36 was used alone in 8 of the 29 studies in that review, the PDQ-39 was used alone in 16 studies, and both were used in 3 of the studies. Moreover, both instruments are recommended by the Movement Disorder Society [11, 12].

Comparing the SF-36 and the PDQ-39 to understand their characteristics is important for deciding whether to use one or both and for understanding the results from each instrument. The relationship between these two instruments has been previously reported [13, 14]. High correlations were reported for the mobility domain of the PDQ-39 and the physical functioning domain in the SF-36, for the emotional well-being (PDQ-39) and mental health (SF-36), and for bodily discomfort (PDQ-39) and bodily pain (SF-36). However, low correlations between the activities of daily living (ADL) (PDQ-39) and role limitations caused by physical problems (SF-36), and between social support (PDQ-39) and social functioning (SF-36), might suggest that those domains actually measure different constructs, although they appear to be associated. Moreover, some special domains in the PDQ-39, such as stigma, cognition, and communication, have no comparable domains in the SF-36 and thus these two instruments may reflect different aspects of HRQoL in patients with PD.

The SF-36 and the PDQ-39 are also likely to be associated with different determinants. In the studies examined in the systematic review [5], predictors of HRQoL included demographic characteristics (age, sex, and employment status, etc.), disease characteristics (disease duration, severity, etc.), motor symptoms (functional ability, etc.), and non-motor symptoms (depression, fatigue, cognition impairment, etc.). Among those factors, depression was consistently reported as a significant determinant in the final model of HRQoL. However, other factors, like age, disease severity, and fatigue, were significant determinants in some, but not in all of the studies that examined these factors. A possible reason for the inconsistent findings across different studies is the different clusters of predictors analyzed and different outcome measures used.

We hypothesized that the significant determinants would depend upon the HRQoL instrument used. The purpose of this study was to identify determinants of HRQoL in patients with PD by using the same set of predictors for the SF-36 and the PDQ-39. The potential determinants examined included demographic and disease characteristics, and motor and non-motor symptoms.

## Methods

### Participants

In this cross-sectional study, 92 participants were recruited consecutively from the neurology departments of two medical centers in southern Taiwan. The inclusion criterion was a diagnosis of PD by a neurologist according to the United Kingdom PD Society Brain Bank criteria [15]. Patients were excluded if they had dementia (Saint Louis University Mental Status Examination [SLUMS] scores < 19 for people with less than a high school education and < 20 for those with a high school education and above) [16]. This study followed the principles of the Declaration of Helsinki. The study protocol was approved by the Institutional Review Board of the hospitals (approval number: B-ER-101-171, EMRP-102-024). Written informed consent was obtained from each participant.

### Procedures and measures

All participants were interviewed face-to-face by one interviewer with the following instruments in a random order. Data were collected from May 2013 to July 2014. Most participants (74/92, 80.4%) were evaluated when they were in the “on” phase of the medication cycle.

Motor and non-motor symptoms were measured using the Movement Disorder Society Revision of the Unified Parkinson’s Disease Rating Scale (MDS-UPDRS) [17], which includes Part I: Non-motor difficulties of daily living (13 items); Part II: Motor difficulties of daily living (13 items); Part III: Motor signs (33 items); and Part IV: Motor complications (6 items). Each item is scored from 0 (normal) to 4 (severe) and item scores are summed for the total score of each part. The Traditional Chinese version of the MDS-UPDRS has been validated in Taiwan and Hong Kong [18].

In addition, non-motor symptoms of depression, fatigue, and cognitive dysfunction were measured using the Geriatric Depression Scale (GDS) [19], the Fatigue Severity Scale (FSS) [20], and the SLUMS, respectively. The 30-item GDS is an efficient depression screening tool for patients with PD [19]. Each item is scored as either 0 or 1. A total score of 10–19 is regarded as mild depression, and 20–30 as severe depression [19]. The Chinese version of the GDS has been reported to have adequate reliability and validity [21].

The 9-item FSS is a unidimensional generic fatigue rating scale that includes items on physical fatigue, mental fatigue, and social aspects [20]. Each item in the FSS is scored from 1 (disagree) to 7 (agree). A mean score ≥ 4 indicates significant fatigue. The FSS has shown good reliability, validity, and sensitivity to changes in patients with PD [20]. The Chinese version of the FSS has been reported to have acceptable reliability and validity in patients with major depressive disorder and non-depressive people [22].

The SLUMS is 30-point screening questionnaire that includes four domains: orientation, memory, attention, and executive functions. The cutoff score for people with less than a high school education is 19 for dementia and 24 for a mild neurocognitive disorder. For those with a high school or higher education, the cutoff score is 20 for dementia and 26 for a mild neurocognitive disorder [16]. The SLUMS has been culturally adapted and translated into Chinese for use in Taiwan [23].

The SF-36 and PDQ-39 were used to measure HRQoL. The SF-36 includes eight domains: physical functioning, role limitations due to physical problems, bodily pain, general health perceptions, mental health, role limitations due to emotional problems, social functioning, and vitality. Eight domain scores and a summary score can be derived (range: 0–100), with higher scores indicating better HRQoL. The Chinese version of the SF-36 has been reported to have good reliability and validity [24].

The PDQ-39 has eight domains: mobility, ADL, emotional well-being, stigma, social support, cognition, communication, and bodily discomfort. It is composed of a single index (PDQ-39 SI) score and eight domain scores (range: 0–100), with higher scores indicating worse HRQoL. Psychometric testing of the Chinese-translated version of the PDQ-39 showed satisfactory reliability and validity in Taiwanese patients with PD [14].

### Statistical analysis

SPSS 17.0 was used for all data analyses. Descriptive statistics were used to summarize the participants’ demographic and clinical characteristics, motor and non-motor symptoms, and HRQoL. Spearman’s rank-correlations were computed for associations between HRQoL and participants’ characteristics and symptoms. Scores of the GDS, FSS and SLUMS were entered as continuous date. Multiple regression analyses were also done using significantly correlated variables to identify the determinants of HRQoL for the SF-36 and for the PDQ-39. Significance was set at *p* < 0.05 (two tailed).

## Results

Of the 92 patients with PD, most (77.2%) were at Hoehn and Yahr (H&Y) stages I and II. Many (65.2%) of them had a mild neurocognitive disorder, and some had depression (23.9%) and fatigue (33.7%) (Table 1). There was no significant difference in the HRQoL scores between those in the “on” and “off” phases of the medication cycle (S1 Table).

**Table 1:** Demographic characteristics and clinical symptoms (N = 92).

Variables	*n* (%)	Mean ± SD	Range
Gender			
Male	60 (65.2)		
Female	32 (34.8)		
Age (years)		65.1 ± 10.11	40–83
Employment status			
Unemployed	56 (60.9)		
Employed	36 (39.1)		
Years of Schooling		9.9 ± 5.00	0–18
Age at Onset		59.3 ± 10.51	34–79
Disease Duration (years)		5.7 ± 4.61	
Hoehn & Yahr Stage			
I	42 (45.6)		
II	29 (31.6)		
III	14 (15.2)		
IV	7 (7.6)		
MDS-UPDRS			
Part I		6.17 ± 4.24	0–22
Part II		9.04 ± 6.40	0–37
Part III		23.96 ± 14.58	3–64
Part IV		2.35 ± 3.66	0–16
SLUMS		24.35 ± 3.37	20–30
Normal	32 (34.8)		
Mild Neurocognitive Disorder	60 (65.2)		
GDS		7.33 ± 6.68	0–28
Normal	70 (76.1)		
Mildly Depressed	13 (14.1)		
Severely Depressed	9 (9.8)		
FSS		3.08 ± 1.66	1–7
Normal	61 (66.3)		
Significant Fatigue	31 (33.7)		
SF-36		62.94 ± 20.08	18–99
PDQ-39		15.29 ± 12.07	0.78–57.19

MDS-UPDRS, Movement Disorder Society-Unified Parkinson’s Disease Rating Scale; SLUMS, Saint Louis University Mental Status Examination; GDS, Geriatric Depression Scale; FSS, Fatigue Severity Scale; SF-36, The 36-item Short Form Health Survey; PDQ-39, The 39-item Parkinson’s Disease Questionnaire.

Employment status, the H&Y stage, the MDS-UPDRS Parts I-III, the GDS, and the FSS were significantly correlated with both the SF-36 and the PDQ-39 (Table 2). In addition, age and the SLUMS were significantly correlated only with the SF-36, but not with the PDQ-39, while disease duration and the MDS-UPDRS Part IV were significantly correlated only with the PDQ-39 but not with the SF-36.

**Table 2:** Correlations of demographics and clinical symptoms with the SF-36 and PDQ-39.

Variables	SF-36	PDQ-39
Gender ^a^	−0.05	0.13
Age	−0.21*	0.05
Employment Status ^b^	0.39**	−0.30**
Years of Schooling	0.09	0.07
Age at Onset	−0.16	−0.07
Disease Duration (years)	−0.09	0.29**
Hoehn & Yahr Stage	−0.36**	0.40**
MDS-UPDRS Part I	−0.60**	0.59**
MDS-UPDRS Part II	−0.65**	0.68**
MDS-UPDRS Part III	−0.34**	0.26**
MDS-UPDRS Part IV	−0.18	0.28*
SLUMS	0.27**	−0.15
GDS	−0.68**	0.64**
FSS	−0.49**	0.41**

^a^ Gender was coded as 0 for female and 1 for male.

^b^ Employment Status was coded as 0 for unemployed and 1 for employed.

MDS-UPDRS, Movement Disorder Society-Unified Parkinson’s Disease Rating Scale; SLUMS, Saint Louis University Mental Status Examination; GDS, Geriatric Depression Scale; FSS, Fatigue Severity Scale; SF-36, The 36-item Short Form Health Survey; PDQ-39, The 39-item Parkinson’s Disease Questionnaire.

* *p* < 0.05,

** *p* < 0.01 (all *p* are two-tailed).

To use the same set of predictors for the multiple regression analyses, we selected the variables that were significantly correlated with both the SF-36 and the PDQ-39 as predictors, including employment status, the H&Y stage, the MDS-UPDRS Parts I-III, the GDS, and the FSS. The SF-36 and the PDQ-39 were separately entered as the outcome variable. The Kolmogorov-Smirnov test was used to check the normality of the SF-36 total scores and the PDQ-39 SI. The test results indicated non-normality for the PDQ-39 SI and thus the logarithm transformation was used.

The results of regression analyses indicated that significant determinants for the SF-36 were the MDS-UPDRS Parts I and II, the GDS, and the FSS, all of which accounted for 61.8% of the variance in the SF-36 (Table 3). The significant determinants for the PDQ-39 were the MDS-UPDRS Parts II and III, and the GDS, which accounted for 54.0% of the variance in the PDQ-39.

**Table 3:** Results of multiple regression analysis.

Predictors	SF-36		PDQ-39	
	standardized coefficients	*p*-value	standardized coefficients	*p*-value
Employment Status^a^	0.083	0.268	0.073	0.376
Hoehn & Yahr Stage	−0.078	0.384	0.192	0.052
MDS-UPDRS Part I	−0.195*	0.032	0.146	0.141
MDS-UPDRS Part II	−0.268*	0.018	0. 466***	0.000
MDS-UPDRS Part III	0.023	0.821	−0.249*	0.026
GDS	−0.281**	0.004	0.288**	0.007
FSS	−0.223**	0.008	0.083	0.364
*R*^*2*^	0.647		0.575	
Adjusted *R*^*2*^	0.618		0.540	
*F* value	22.01***		16.24***	

^a^ Employment Status was coded as 0 for unemployed and 1 for employed.

MDS-UPDRS, Movement Disorder Society-Unified Parkinson’s Disease Rating Scale; GDS, Geriatric Depression Scale; FSS, Fatigue Severity Scale; SF-36, The 36-item Short Form Health Survey; PDQ-39, The 39-item Parkinson’s Disease Questionnaire.

* *p* < 0.05,

** *p* < 0.01,

*** *p* < 0.001 (all *p* are two-tailed).

We further conducted correlational analyses to understand the relationship between the HRQoL domains and the significant determinants (i.e., the MDS-UPDRS Parts I-III, the GDS, and the FSS) (Table 4). The GDS was significantly correlated with all domains in each instrument. The MDS-UPDRS Parts I and II and the FSS were correlated with all domains in the SF-36 and most in the PDQ-39. The MDS-UPDRS Part III was correlated with the physical functioning, role-physical, bodily pain, general health, and vitality domains in the SF-36 and the mobility, ADL, and bodily discomfort domains of the PDQ-39.

**Table 4:** Correlations between domains of HRQoL and significant determinants.

Variables	MDS-UPDRS Part I	MDS-UPDRS Part II	MDS-UPDRS Part III	GDS	FSS
**SF-36**					
Physical functioning	−0.38**	−0.70**	−0.52**	−0.35**	−0.19
Role-physical	−0.41**	−0.56**	−0.22*	−0.54**	−0.35**
Bodily pain	−0.38**	−0.38**	−0.22*	−0.27*	−0.31**
General health	−0.43**	−0.39**	−0.26*	−0.28**	−0.23*
Mental health	−0.54**	−0.31**	−0.16	−0.65**	−0.42**
Role-emotional	−0.33**	−0.36**	−0.15	−0.48**	−0.38**
Social functioning	−0.41**	−0.47**	−0.14	−0.47**	−0.32**
Vitality	−0.60**	−0.42**	−0.24*	−0.74**	−0.63**
Total scores	−0.60**	−0.65**	−0.34**	−0.68**	−0.49**
**PDQ-39**					
Mobility	0.51**	0.73**	0.37**	0.49**	0.35**
ADL	0.32**	0.65**	0.30**	0.39**	0.13
Emotional well-being	0.50**	0.38**	0.06	0.59**	0.51**
Stigma	0.20	0.24*	−0.07	0.45**	0.20
Social support	0.32**	0.17	−0.02	0.57**	0.43**
Cognition	0.44**	0.29**	0.03	0.34**	0.29**
Communication	0.35**	0.38**	0.15	0.37**	0.36**
Bodily discomfort	0.44**	0.43**	0.26*	0.29**	0.26*
Summary index	0.59**	0.67**	0.25*	0.64**	0.41**

MDS-UPDRS, Movement Disorder Society-Unified Parkinson’s Disease Rating Scale; GDS, Geriatric Depression Scale; FSS, Fatigue Severity Scale; SF-36, The 36-item Short Form Health Survey; PDQ-39, The 39-item Parkinson’s Disease Questionnaire.

* *p* < 0.05,

** *p* < 0.01 (all *p* are two-tailed).

## Discussion

By using the same cluster of predictors for the SF-36 and the PDQ-39, we found that depressive symptoms and motor difficulties of daily living were the first two significant determinants for both instruments. The other significant determinant for the SF-36 was fatigue and non-motor difficulties of daily living and for the PDQ-39 was motor signs.

Our finding that depression was a major determinant in the SF-36 and the PDQ-39 are consistent with other studies and reviews [5,25–30]. We found that depression was significantly correlated with not only psychosocial but also physical functioning domains in the SF-36 and the PDQ-39. Similar results were also reported in a recent study [31] that depression was significantly correlated with most domains of the PDQ-39 and was a significant determinant for the mobility, emotional well-being, stigma, and communication domains. Using mediation analysis, research [32] reported that depression might contribute to ADL difficulties, which in turn might contribute to decreased HRQoL in patients with PD. The interrelationships among depression, physical function, and HRQoL suggest the need for an interdisciplinary approach to alleviate the effects of psychological and physical dysfunction on the HRQoL of patients with PD.

The MDS-UPDRS Part II was another significant determinant in both the SF-36 and the PDQ-39. Motor difficulties of daily living were associated with poor HRQoL, which is consistent with other studies using the PDQ-39 [28] and other HRQoL tools (e.g., EQ-5D, PDQ-8) [33, 34]. The MDS-UPDRS Part II asks questions directly related to daily activities with patients’ views of primary disease symptoms, and it has been widely used to assess motor symptoms and disabilities in PD [33–35]. Our finding that the MDS-UPDRS Part II was more strongly associated with the HRQoL instruments than were the MDS-UPDRS Part III and the H&Y stage is also consistent with other research [28], which suggests that the role of the motor difficulties of daily living outweighs the roles of motor symptoms and disease severity in HRQoL. The difficulties completing simple daily tasks (e.g., using eating utensils, dressing, and walking) adversely affect HRQoL in patients with PD [5, 32].

Fatigue was also a significant determinant for the SF-36, which is congruent with one review [5] and two studies using the SF-36 [36, 37]. Many domains of the SF-36 encompass attributes of energy, strength, and endurance. For example, the domain of physical functioning asks questions about vigorous and moderate activities, and the domain of role limitations due to physical problems asks about overall activity performance in daily life in terms of time and types. Therefore, the association between the SF-36 and the FSS might be attributable to the similar properties that underlie the questionnaire items.

In addition, the non-motor difficulties of daily living (the MDS-UPDRS Part I) were also a significant determinant for the SF-36. The MDS-UPDRS Part I evaluates a broad range of non-motor symptoms, such as anxious mood, apathy, features of dopamine dysregulation syndrome, daytime sleepiness, and urinary problems. Our results are consistent with recent findings [38–40] that the overall burden of non-motor symptoms was a significant contributor to HRQoL in PD, suggesting the importance of active screening and managing non-motor symptoms.

In agreement with other research [41], we found that motor signs (the MDS-UPDRS Part III) were also a significant determinant for the PDQ-39. The results suggest that the mobility, ADL, and bodily discomfort domains of the PDQ-39 are also related to motor signs of PD in addition to motor difficulties of daily living. PD motor features are frequently evaluated to help healthcare professionals understand the response to dopaminergic therapy [42]. Our findings suggest that the PDQ-39 may be a more responsive tool than the SF-36 for reflecting HRQoL changes related to motor signs.

Our findings show that the SF-36 and the PDQ-39 reflect somewhat different aspects of the lives of patients with PD. Specifically, the SF-36 is more associated with non-motor symptoms, and the PDQ-39 is more related to the motor symptoms of PD. Therefore, it is important to know what HRQoL instrument is used to analyze the contributing factors of HRQoL, rather than assuming that all HRQoL instruments reflect the same construct. The choice of which instrument to use may depend upon how much the area of interest is related to general functioning or to PD-related symptoms. It was recently suggested [5, 12] that both generic and disease-specific measures be used to capture unexpected improvement. However, considering the extended time and effort that would be needed with more evaluations, we suggest that an understanding of the HRQoL instrument’s characteristics is important.

In addition, we noticed that the stigma domain in the PDQ-39 was significantly associated with only depression and motor difficulties of daily living, while most of the other HRQoL domains were associated with more than three of the significant determinants. The PDQ-39 was developed from in-depth interviews with patients with PD to learn about the areas of their lives that had been adversely affected by their PD [10]. Therefore, the domains in the PDQ-39 reflect the concerns of patients with PD and should be carefully examined. The finding that the stigma domain had only scant correlations with PD symptoms suggests that merely addressing motor and non-motor symptoms may not be enough, and that a broader consideration from the social context is needed to understand stigma in PD.

This study has some limitations. Our participants were recruited from medical centers, and thus the generalizability may be limited to clinic-based patients with PD. In addition, we did not collect information on medications or levodopa equivalent daily lose, or control for the medication cycle phase during the evaluation. Future research should include medication information and be consistent on the phase of medication cycle during the evaluation. This is a cross-sectional study in which all assessments were administered at one time point. Follow-up of the participants would be helpful for understanding the longitudinal change and to explore possible causal relationships between participants’ characteristics and HRQoL. Finally, the determinants we examined were factors related to the individual. Future work should include physical and social environmental factors to broaden our understanding of the determinants of HRQoL in patients with PD.

## Conclusions

This is the first study that uses the same set of predictors for both commonly used generic and disease-specific HRQoL instruments in PD. The common determinants in both the SF-36 and PDQ-39 suggest the importance of evaluating and intervening on depressive symptoms and motor difficulties of daily living in patients with PD. In addition, we found that the SF-36 seems to be more related to non-motor symptoms, while the PDQ-39 appears more related to motor symptoms. This information is important for choosing which instrument to use in clinical practice and for research purposes.

## Supporting information

S1 Table Comparison of HRQoL scores between patients with PD in the “on” and “off” phases of the medication cycle. (PDF)

S1 Appendix Raw data of all relevant data in all participants. (XLSX)
