# Supplementary material for: Determinants of generic and specific health-related quality of life in patients with Parkinson’s disease
Source: PLoS One. 2017 Jun 26;12(6):e0178896. doi: 10.1371/journal.pone.0178896 (PMC5484474; doi:10.1371/journal.pone.0178896)
Supplement: S1 Table — (PDF) [file pone.0178896.s001.pdf]

S1 Table. Comparison of HRQoL scores between patients with PD in the "on" and "off" phases of the medication cycle.

Variables	Off phase (n = 18) Mean rank	On phase (n = 74) Mean rank	Z value	*p*-value†
**SF-36**				
Physical functioning	52.36	45.07	–1.041	0.298
Role-physical	49.86	45.68	–0.610	0.542
Bodily pain	53.78	44.73	–1.341	0.180
General health	36.97	48.82	–2.691	0.091
Mental health	51.33	45.32	–0.859	0.390
Role-emotional	45.08	46.84	–0.266	0.790
Social functioning	44.61	46.96	–0.343	0.732
Vitality	52.69	44.99	–1.099	0.272
Total scores	47.81	46.18	–0.231	0.817
**PDQ-39**				
Mobility	45.03	46.86	–0.262	0.793
ADL	51.86	45.20	–0.965	0.335
Emotional well-being	48.11	46.11	–0.289	0.772
Stigma	43.53	47.22	–0.553	0.581
Social support	44.64	46.95	–0.427	0.669
Cognition	44.72	46.93	–0.318	0.751
Communication	47.36	46.29	–0.172	0.864
Bodily discomfort	37.03	48.80	–1.702	0.089
Summary index	43.75	47.17	–0.487	0.626

SF-36, The 36-item Short Form Health Survey; PDQ-39, The 39-item Parkinson's Disease Questionnaire.

† Using the Mann–Whitney test.
